# Osgood-Schlatter Disease Awareness and Prevalence Among Individuals Aged 10-25 in Saudi Arabia: A Cross-Sectional Study

**DOI:** 10.7759/cureus.81299

**Published:** 2025-03-27

**Authors:** Daifallah Mohammed Alharbi, Abdullah M Alraddadi, Abdulaziz Mohammed R Alraddadi, Emad A Alsaedi, Nawaf Mohammed S Alotaibi, Abdulmalik B Albaker

**Affiliations:** 1 Department of Orthopaedics, College of Medicine, Majmaah University, Majmaah, SAU; 2 College of Medicine, Majmaah University, Majmaah, SAU; 3 Department of Medicine and Surgery, Al-Rayan National College of Medicine, Madinah, SAU; 4 Department of Internal Medicine, King Salman Bin Abdulaziz Medical City, Madinah, SAU

**Keywords:** adolescents, awareness, osgood-schlatter disease, prevalence, saudi arabia

## Abstract

Background

Osgood-Schlatter Disease (OSD) is a skeletal pathology primarily affecting young adults. It is characterized by anterior knee pain and limited mobility due to repetitive quadriceps contractions. Severe cases can lead to chronic knee pain and tendinosis. This study investigates the prevalence, awareness, and impact of OSD among Saudi Arabian youth.

Methods

A cross-sectional survey was conducted among 275 participants aged 10-25 years. Participants completed a self-administered questionnaire for demographic details, the Knee and Osteoarthritis Outcome Score (KOOS)-child scale for awareness and opinions, anthropometric measurements, and a physical examination of symptoms. Data were analyzed using IBM SPSS Statistics for Windows, Version 27 (released 2020; IBM Corp., Armonk, NY, USA).

Results

The sample comprised 59.6% males (n = 164) and 40.4% females (n = 111), with 52.7% (n = 145) aged 10-15 years and 47.3% (n = 130) older than 15 years. The majority of participants reported pain attacks without pain in between (30.9%, or n = 85) or constant pain with slight fluctuations (26.2%, or n = 72). The KOOS subscales revealed moderate knee-related problems across all dimensions: Painful (M = 52.9, SD = 20.9), Difficulty With Daily Activity (M = 50.5, SD = 22.3), Difficulty During Exercise and Play (M = 49.4, SD = 23.9), Knee Problems (M = 58.0, SD = 19.2), and Impact on Quality of Life (M = 56.6, SD = 20.8). Females reported significantly worse outcomes than males in Painful (p = 0.005), Difficulty With Daily Activity (p = 0.001), Difficulty During Exercise and Play (p = 0.022), and Impact on Quality of Life (p = 0.037). No significant differences were found between age groups in any KOOS subscales.

Conclusion

These findings highlight the significant burden of OSD in terms of pain severity and functional limitations, and identify important gender differences in OSD outcomes, underscoring the need for targeted interventions to improve quality of life. These findings call for proactive clinical and community-based interventions to mitigate OSD’s impact on Saudi youth, with special attention to females and active adolescents.

## Introduction

Osgood-Schlatter Disease (OSD), also referred to as Lannelongue disease, is a form of osteochondrosis first described by Osgood and Schlatter in 1903 [1]. It is characterized by traction apophysitis resulting from repetitive contractions of the quadriceps femoris muscle [2]. Approximately 9.8% of adolescents are affected by OSD, with a higher prevalence among those who participate in high-risk sports [1,3]. Research indicates that approximately 40% of patients experience persistent discomfort, which can lead to chronic knee pain and tendinosis, potentially affecting their quality of life [1,4]. Moreover, OSD has been associated with various conditions, including compartment syndrome, meniscal and patellar tendon injuries, hyperactivity, and attention deficit disorders [1].

Morphological changes in the patella and its tendon, vascular insufficiency of the tibial apophysis, and angular changes in the knee are also linked to OSD. Sports involving high-intensity impact, repetitive muscle-tendon loading, and frequent exercise, such as basketball, soccer, and volleyball, are significant contributors to the development of OSD. Poorly supervised sports practices, often conducted under inadequate training conditions, can exacerbate traumatic and degenerative processes in the musculoskeletal systems of adolescents. These activities frequently coincide with the pubertal growth phase, during which accelerated bone growth, due to hormonal changes, increases the risk of OSD in physically active adolescents [3]. This pubertal growth phase involves the secondary ossification center of the anterior tibial tuberosity, which typically forms around the age of 9 in girls and 11 in boys. Thus, symptoms often manifest between the ages of 8 and 12 in girls and 12 and 15 in boys, though cases in adults have also been reported [2]. The most common symptom of OSD is pain and tenderness over the tibial tuberosity. OSD develops gradually and is commonly associated with repetitive knee movements, with tenderness over the tibial tubercle being a hallmark feature [2]. Non-traumatic knee pain is the most frequent cause of pain in adolescents, with patellofemoral pain (PFP) and OSD being the most prevalent conditions [5]. Both PFP and OSD share similar pain characteristics, such as anterior thigh pain, and are particularly common among highly active teenagers [5,6].

While a diagnosis can often be made through patient history and physical examination, imaging techniques (radiographs, ultrasounds, CT scans, and MRI) are frequently employed to assess tibial tuberosity abnormalities and rule out other conditions [7]. Treatment typically involves symptomatic relief through ice application and non-steroidal anti-inflammatory drugs (NSAIDs), activity modification, relative rest from aggravating activities, and lower extremity stretching exercises to address underlying biomechanical issues [1,4]. Although OSD is a benign condition, recovery can be prolonged, often resulting in time away from sports.

In Saudi Arabia, a study conducted in Majmah showed that PFP was identified in 94 individuals (38.8%), with females accounting for 72.3% of cases and males for 27.7%. The study found that age was a significant predictor of PFP, while gender and BMI were not [8]. Another study involving 676 Saudi adolescents aged 10-18 years found a significantly higher prevalence of knee pain (26%) compared to males (19.2%). Age and BMI were identified as significant predictors of knee pain, with a significant correlation between BMI classification and knee stiffness (p = 0.008), as well as between being engaged in recreational physical activities and difficulty bending their knees (p = 0.03) [9].

While studies have highlighted the prevalence of musculoskeletal problems, particularly knee pain, among Saudi adolescents, there remains a lack of awareness and research specifically targeting OSD in this population. To date, no study in Saudi Arabia has directly investigated the prevalence and awareness of OSD. Therefore, this study aims to address this gap by exploring the prevalence and awareness of OSD among individuals aged 10-25 years in Saudi Arabia.

## Materials and methods

Study design and setting

This cross-sectional study was conducted from November 2023 to May 2024 in the Kingdom of Saudi Arabia to investigate the prevalence and awareness of OSD among adolescents and young adults. The study aimed to provide insights into the demographic and clinical characteristics of OSD in this population.

Study population

The study population included both male and female adolescents and young adults aged 10-25 who resided in Saudi Arabia without neurologic abnormalities. Though OSD is mainly an adolescent disease, the literature indicates that symptoms may endure in some individuals into early adulthood [1,2], and participants up to 25 years old were targeted in order to capture cases with long residual symptoms, late resolution, or misdiagnosis. Inclusion criteria were participants aged between 10 and 25 who resided in Saudi Arabia without neurological abnormalities. We excluded participants with neurological abnormalities, those outside the age range of 10-25, and non-residents of Saudi Arabia.

Sample size and sampling technique

The sample size was calculated using the WHO sample size calculator, resulting in a required sample of 279 Saudi Arabs, both males and females, with a 95% confidence interval. A convenient, non-probability sampling technique was employed to recruit participants. This technique is convenient because most potential participants were school students or young athletes, making it difficult to implement probability-based recruitment.

Data collection method and tool

The study utilized a self-administered questionnaire to collect demographic information, including gender, age, puberty status, and participation in sports. Additionally, the Knee and Osteoarthritis Outcome Score (KOOS)-child scale [10] was used to evaluate participants' awareness and understanding of knee-related issues. The KOOS-child scale is a standardized tool designed to assess knee-related problems in children and adolescents. It is not an original questionnaire developed for this study, but rather a well-established instrument used in clinical and research settings to measure knee health and function.

The Arabic-translated questionnaire underwent pilot testing before full implementation to ensure validity and reliability. Bilingual experts translated the questionnaire from English to Arabic and then back-translated it to ensure content accuracy. Then, a panel of physiotherapists and sports medicine specialists reviewed the questionnaire to ensure clarity and relevance. Pilot testing was also conducted with a small group of 30 participants (excluded from the final analysis) to assess comprehension, wording clarity, and the time required to complete the survey.

Researchers approached public and private schools across multiple regions in Saudi Arabia, inviting students to participate in school health screenings or physical education sessions. They also approached families in public walkways and recreational areas (e.g., parks) to recruit participants. Then, a web-based survey was disseminated via parent/student social media groups to reach geographically dispersed participants.

Pain score calculation/evaluation

The pain score was calculated and evaluated using the KOOS-child scale, which includes several subscales to assess different dimensions of knee health. The subscales relevant to pain and functional limitations are: (1) Painful: evaluates the severity of pain experienced by the participant; (2) Difficulty With Daily Activity: assesses the impact of knee pain on daily activities; (3) Difficulty During Exercise and Play: measures the limitations experienced during physical activities; (4) Knee Problems: evaluates the overall knee-related issues; (5) Impact on Quality of Life: assesses how knee pain affects the participant's quality of life.

Participants' responses to each item within the five KOOS subscales were assigned scores ranging from 0 to 4, where 0 represents no pain or limitations and 4 represents severe pain or limitations. The raw score for each subscale was calculated by summing the individual item scores. The raw scores were then transformed into a normalized score ranging from 0 to 100, where 0 indicates the most severe symptoms or functional limitations and 100 represents no symptoms or limitations. Higher scores on the normalized scale indicate better knee-related outcomes.

Pain intensity ratings

Participants were also asked to rate their current pain intensity, peak pain intensity over the past six months, and average pain severity over the past six months using a numerical scale (likely from 0 to 10, where 0 is no pain and 10 is the worst pain imaginable). These pain ratings were used to assess the severity of pain and its impact on the participants' daily lives.

Physical examination

The physical examination was conducted by a single physical therapist to maintain consistency and reliability in the assessment process. This examination included a thorough inspection and palpation of the tibial tuberosity and knee region to identify any abnormalities. Discomfort in the tibial tuberosity was assessed to rule out tibial tuberosity apophysitis, while tenderness or pain at the tibial tubercle was evaluated as a key diagnostic criterion for OSD. Participants were also observed during various activities, such as walking, running, jumping, or kneeling, to detect any pain or discomfort that could be associated with OSD.

Anthropometric measurements were taken to gather objective data on the participants' physical characteristics. Weight was measured using an electronic scale, and height was determined using a handheld stadiometer. These measurements provided essential data for analyzing the relationship between physical attributes and the prevalence of OSD. Together, these methods ensured a holistic approach to data collection, enabling a detailed and accurate understanding of the condition among the study population.

Training for physical therapists

The physical therapist underwent specific training to ensure consistent and reliable assessments. They participated in standardization sessions and inter-rater reliability testing and used structured assessment protocols. A checklist was developed to reduce subjective variability, and ongoing quality control involved randomized cross-checking of a subset of examinations by another specialist.

Data analysis

Data were analyzed using IBM SPSS Statistics for Windows, Version 27 (released 2020; IBM Corp., Armonk, NY, USA), and visualization was performed using Microsoft Excel 365 (Microsoft® Corp., Redmond, WA, USA). Quantitative data were reported using mean, standard deviation, minimum, and maximum, whereas qualitative data were described using numbers and percentages (%). The normalized KOOS subscale scores were used for subsequent statistical analyses, with higher scores indicating better knee-related outcomes for each subscale. Subsequently, the independent sample t-test was used to compare the subscale scores across gender and age categories. In addition, Spearman's rho correlation analyses were used to determine the relationships between different pain ratings and KOOS subscale scores. Values less than 0.05 were judged to be statistically significant, and values less than 0.01 were considered highly significant.

Ethical considerations

Ethical clearance was obtained from the Ethical Review Board of Majmaah University for Research Ethics Committee (MUREC-Sep.17/COM-2023/28-5). Participants were informed about the research purpose, and their consent was obtained. The questionnaire and consent forms were provided in Arabic to ensure comprehension. The confidentiality of participants' personal data was strictly maintained throughout the study.

## Results

Socio-demographic and clinical characteristics of the participants

Table 1 presents the socio-demographic and clinical characteristics of the 275 participants with OSD. The sample consisted of 59.6% males (n = 164) and 40.4% females (n = 111). Regarding age, 52.7% (n = 145) were between 10 and 15 years old, while 47.3% (n = 130) were older than 15 years. The majority of participants reported experiencing pain attacks without pain in between (30.9%, or n = 85) or constant pain with slight fluctuations (26.2%, or n = 72). Most participants (88.7%, or n = 244) did not report pain spreading to other areas of the body.

**Table 1:** Demographic and Clinical Characteristics of Participants With Osgood-Schlatter Disease (N = 275)

Variables	N	%
Sex		
Female	111	40.40%
Male	164	59.60%
Age (years)		
From 10-15	145	52.70%
Older than 15	130	47.30%
Course of the pain		
There is no persistent pain	5	1.80%
The pain is constant, with slight fluctuations	72	26.20%
Persistent pain with bouts of pain	57	20.70%
Pain attacks without pain between them	85	30.90%
Pain attacks with pain in between	56	20.40%
Pain spread to other areas of the body		
No	244	88.7%
Yes	31	11.3%

**Figure 1:**
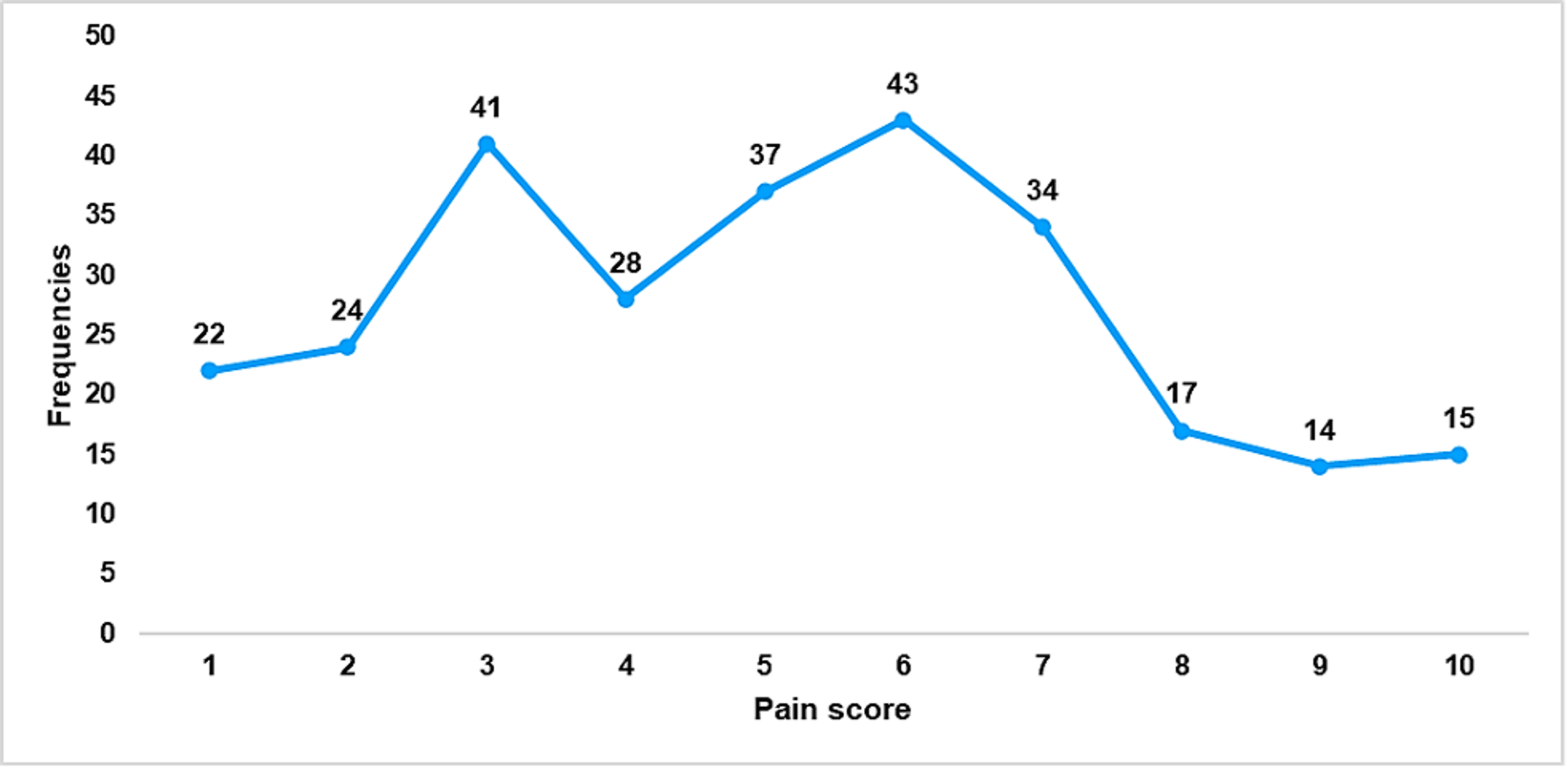
Distribution of Current Pain Intensity Ratings in Participants With Osgood-Schlatter Disease

In addition, Figure 1 illustrates the distribution of current pain intensity ratings reported by participants with OSD at the time of assessment. The most commonly reported pain scores were 6 (43 participants) and 3 (41 participants), followed by 5 (37 participants) and 7 (34 participants). Lower pain scores, such as 1 (22 participants) and 2 (24 participants), were reported less frequently. A decreasing trend was observed at higher pain scores, with 8 (17 participants), 9 (14 participants), and 10 (15 participants).

**Figure 2:**
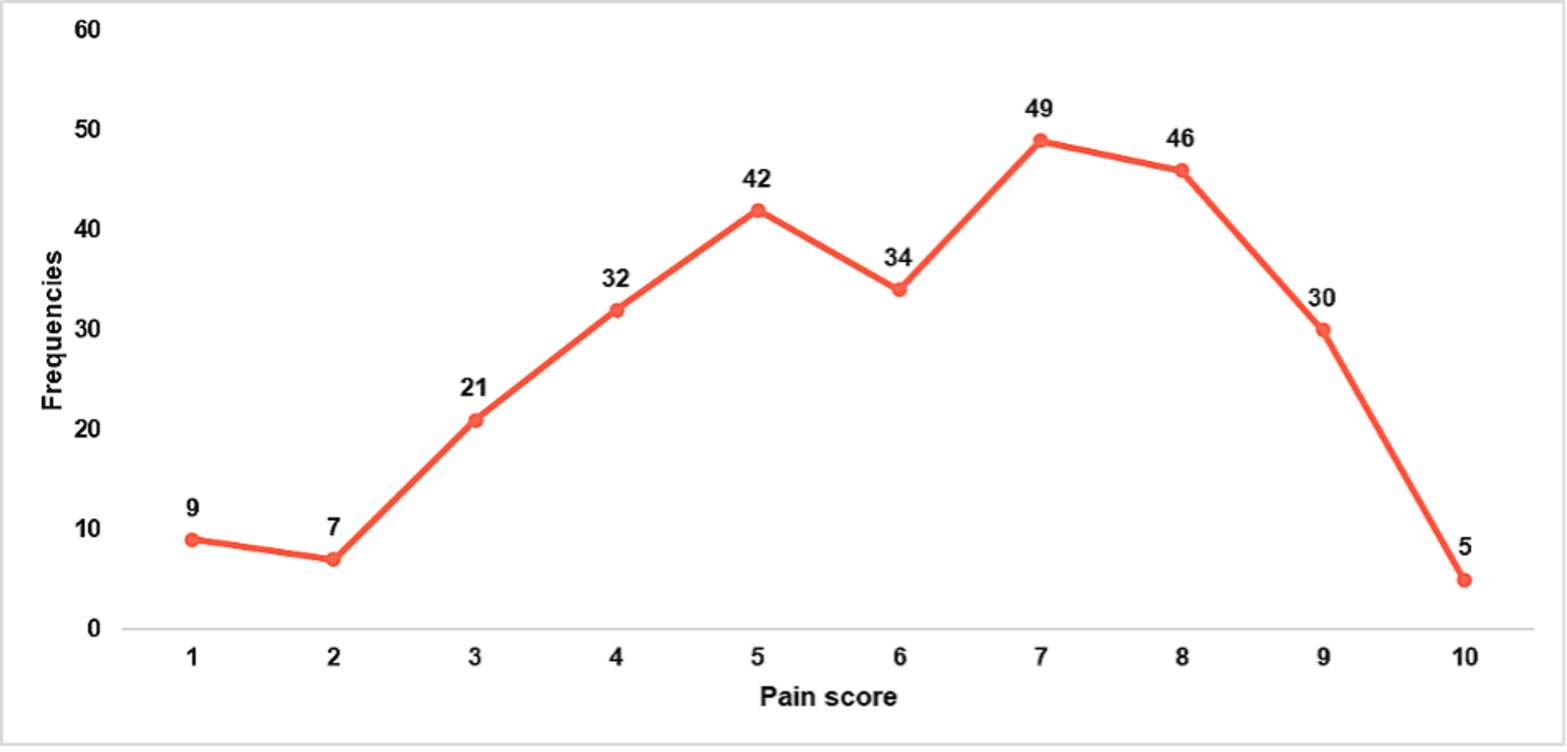
Distribution of Peak Pain Intensity Ratings in the Past 6 Months Among Participants With Osgood-Schlatter Disease

Moreover, Figure 2 presents the distribution of peak pain intensity ratings experienced by participants with OSD over the past six months. The most frequently reported pain score was 7 (49 participants), followed by 8 (46 participants) and 5 (42 participants). Moderate pain levels (scores 4-6) were also common, with 32 participants reporting a score of 4 and 34 participants reporting a score of 6. Lower pain scores (1-3) were reported less frequently, with nine participants at a score of 1, seven participants at a score of 2, and 21 participants at a score of 3. The frequency of extreme pain scores (9-10) declined sharply, with 30 participants reporting a score of 9 and only five participants reporting the highest pain score of 10.

**Figure 3:**
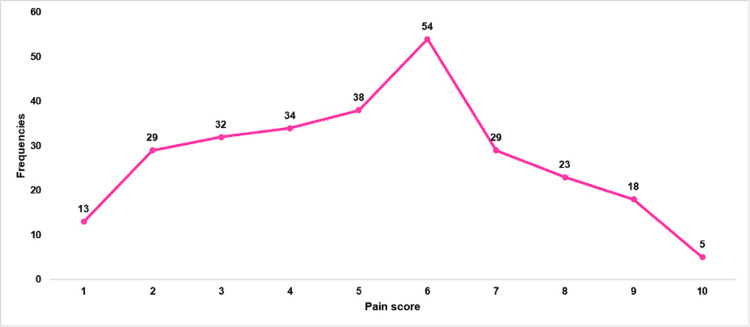
Distribution of Average Pain Severity Ratings Over the Past 6 Months Among Participants With Osgood-Schlatter Disease

Figure 3 presents the distribution of average pain severity ratings experienced by participants with OSD over the past six months. The most frequently reported pain score was 6 (54 participants), followed by 5 (38 participants) and 4 (34 participants). Pain scores of 3 and 2 were also commonly reported, with 32 participants and 29 participants, respectively. Lower pain scores (1) were reported by 13 participants, while higher pain scores showed a decreasing trend, with 29 participants at a score of 7, 23 participants at a score of 8, 18 participants at a score of 9, and only five participants at the highest pain score of 10.

KOOS subscale findings

Table 2 displays the descriptive statistics for the KOOS subscales. The mean scores for all subscales were: Painful (M = 52.9, SD = 20.9), Difficulty With Daily Activity (M = 50.5, SD = 22.3), Difficulty During Exercise and Play (M = 49.4, SD = 23.9), Knee Problems (M = 58.0, SD = 19.2), and Impact on Quality of Life (M = 56.6, SD = 20.8). All these subscales fell within the moderate level range, indicating moderate knee-related problems across all dimensions assessed by the KOOS in this sample. The range of scores (minimum to maximum) varied across subscales, demonstrating a variety of experiences within the participant group.

**Table 2:** Descriptive Statistics for KOOS Subscales in Participants With Osgood-Schlatter Disease (N = 275) Level scores: from 0-40, low; from 40.1-60, moderate; and from 60.1-100, high KOOS: Knee and Osteoarthritis Outcome Score

Subscales	Minimum	Maximum	Mean	SD	Level
Painful	3.57	100.0	52.9	20.9	Moderate
Difficulty with daily activity	4.55	100.0	50.5	22.3	Moderate
Difficulty during exercise and play	0.00	100.0	49.4	23.9	Moderate
Knee problems	0.00	100.0	58.0	19.2	Moderate
Impact on quality of life	0.00	100.0	56.6	20.8	Moderate

**Figure 4:**
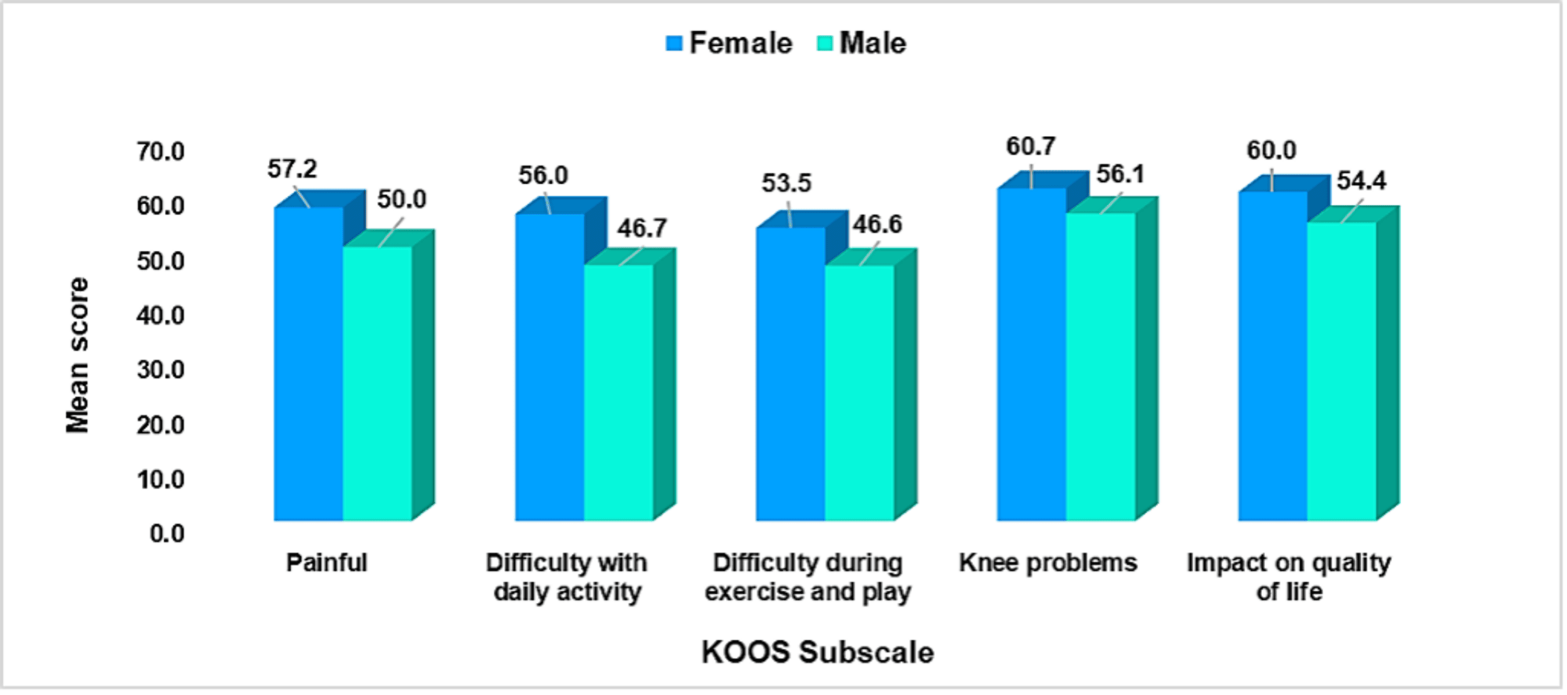
Comparison of KOOS Subscale Scores Between Female and Male Participants With Osgood-Schlatter Disease KOOS: Knee and Osteoarthritis Outcome Score

Table 3 and Figure 4 show the sex differences in KOOS subscale scores. Statistically significant differences were observed in several subscales. Females exhibited significantly higher mean scores, indicating worse outcomes, compared to males in Painful (Females: M = 57.2, SD = 22.9; Males: M = 50.0, SD = 19.1; p = 0.005), Difficulty With Daily Activity (Females: M = 56.0, SD = 24.0; Males: M = 46.7, SD = 20.3; p = 0.001), Difficulty During Exercise and Play (Females: M = 53.5, SD = 25.5; Males: M = 46.6, SD = 22.3; p = 0.022), and Impact on Quality of Life (Females: M = 60.0, SD = 23.8; Males: M = 54.4, SD = 18.3; p = 0.037). No significant difference was found between sexes in the Knee Problems subscale (p = 0.067).

**Table 3:** Independent Samples t-test Comparing KOOS Subscale Scores Between Female and Male Participants With Osgood-Schlatter Disease *Significant at p ≤ 0.05; **Significant at p ≤ 0.01 p-value is calculated by the independent t-test KOOS: Knee and Osteoarthritis Outcome Score

Subscales	Female		Male		p-value
	Mean	SD	Mean	SD	
Painful	57.2	22.9	50.0	19.1	0.005**
Difficulty with daily activity	56.0	24.0	46.7	20.3	0.001**
Difficulty during exercise and play	53.5	25.5	46.6	22.3	0.022*
Knee problems	60.7	21.9	56.1	16.9	0.067
Impact on quality of life	60.0	23.8	54.4	18.3	0.037*

Table 4 presents the comparison of the KOOS subscale scores between younger (10-15 years) and older (older than 15 years) age groups. No statistically significant differences were found between the age groups on any of the KOOS subscales (Painful, Difficulty With Daily Activity, Difficulty During Exercise and Play, Knee Problems, and Impact on Quality of Life; all p > 0.05).

**Table 4:** Independent Samples t-test Comparing KOOS Subscale Scores Between Age Groups (10-15 Years vs. Older Than 15 Years) in Participants With Osgood-Schlatter Disease p-value is calculated by the independent t-test KOOS: Knee and Osteoarthritis Outcome Score

Subscales	From 10-15 years		Older than 15 years		p-value
	Mean	SD	Mean	SD	
Painful	51.1	20.1	54.9	21.7	51.1
Difficulty with daily activity	50.7	21.4	50.2	23.4	50.7
Difficulty during exercise and play	49.3	24.4	49.5	23.3	49.3
Knee problems	58.6	17.9	57.3	20.6	58.6
Impact on quality of life	55.8	20.4	57.6	21.3	55.8

The Spearman's rho correlation analyses were conducted to examine the relationships between different pain ratings and KOOS subscale scores, as shown in Table 5. Significant negative correlations (p < 0.001) were observed between all three pain ratings (Current Pain Level, Peak Pain Level in the Past Six Months, and Average Pain Severity in the Past Six Months) and all five KOOS subscales. Correlation coefficients ranged from -0.418 to -0.680, indicating moderate to strong negative associations. Specifically, higher levels of current pain, peak pain, and average pain severity were significantly associated with lower scores on all KOOS subscales, reflecting worse knee-related outcomes as the pain increased (p < 0.001).

**Table 5:** Spearman's Rho Correlations Between Pain Ratings and KOOS Subscale Scores in Participants With Osgood-Schlatter Disease **Significant at p ≤ 0.01 p-value is calculated using Spearman's rho test KOOS: Knee and Osteoarthritis Outcome Score

Subscales	Current pain level		Peak pain level (past 6 months)		Average pain severity (past 6 months)	
	Correlation coefficient	p-value	Correlation coefficient	p-value	Correlation coefficient	p-value
Painful	-0.484**	<0.001**	-0.587**	<0.001**	-0.566**	<0.001**
Difficulty with daily activity	-0.636**	<0.001**	-0.671**	<0.001**	-0.680**	<0.001**
Difficulty during exercise and play	-0.530**	<0.001**	-0.619**	<0.001**	-0.592**	<0.001**
Knee problems	-0.463**	<0.001**	-0.540**	<0.001**	-0.522**	<0.001**
Impact on quality of life	-0.418**	<0.001**	-0.528**	<0.001**	-0.487**	<0.001**

## Discussion

The findings of this study provide valuable insights into the prevalence, awareness, and impact of OSD among adolescents and young adults aged 10-25 years in Saudi Arabia. The results highlight the OSD prevalence, the severity of pain experienced by participants, and the functional impact of OSD. These findings are consistent with previous research, while also offering new perspectives on the management and understanding of OSD in this population.

The study revealed that OSD primarily affects adolescent males (59.6%), with the most affected age group being 12-18 years. This aligns with existing literature, which indicates that OSD is more prevalent in males due to their higher participation in high-impact sports and physical activities during adolescence [1,4]. The higher prevalence in males may also be attributed to differences in the timing of the pubertal growth spurt, which occurs later in boys than in girls, coinciding with the period of peak physical activity [3]. However, the study also found that females reported significantly worse outcomes in several KOOS subscales, including Pain, Difficulty With Daily Activities, and Impact on Quality of Life. These findings align with previous research [1,11]. This suggests that while males may be more likely to develop OSD, females may experience more severe symptoms and functional limitations once affected. This finding agrees with some previous research in Saudi Arabia, which reported a significant difference in knee pain between males and females, with females reporting significantly more pain than males (p = 0.003) [9].

The mean pain severity over the past six months was 5.15, indicating consistent moderate pain among participants. This finding is consistent with previous studies that have reported moderate to severe pain as a hallmark feature of OSD [2,7,12]. The study also found that 88.7% of participants did not report pain spreading to other areas of the body, suggesting that OSD primarily localizes to the knee region. However, the persistence of pain, as evidenced by the high frequency of pain attacks and constant pain with slight fluctuations, underscores the chronic nature of OSD and its potential to affect long-term musculoskeletal health. The distribution of pain intensity ratings revealed that moderate pain levels (scores 4-6) were most commonly reported, with a decreasing trend at higher pain scores. This pattern aligns with previous research, indicating that while OSD can cause significant discomfort, extreme pain is relatively rare [6]. However, the presence of severe pain in some participants highlights the need for targeted interventions to manage pain effectively and prevent long-term complications, such as chronic tendinosis or patellar tendon injuries [4].

The KOOS subscale findings indicated moderate knee-related problems across all dimensions assessed, including Pain, Difficulty With Daily Activities, Difficulty During Exercise and Play, Knee Problems, and Impact on Quality of Life. These results are consistent with previous studies that have used the KOOS scale to evaluate knee-related outcomes in adolescents with OSD [6,7,13]. The moderate scores suggest that OSD significantly impacts the functional capacity and quality of life of affected individuals, particularly in terms of their ability to engage in physical activities and perform daily tasks. The discrepancy in pain among female vs. male participants may be explained by the likelihood of females reporting pain due to cultural or social factors. Research confirmed that women report pain more frequently than men [14]. Moreover, anatomical differences, such as wider pelvic structures or greater Q-angles, may predispose females to more severe symptoms [3].

The findings of this study are largely consistent with previous research on OSD. For example, the higher prevalence of OSD in males and its association with high-impact sports align with studies [1,4]. Similarly, the moderate pain severity, which impacts the quality of life, is consistent with findings from [6,7]. However, this study adds to the literature by highlighting the significant gender differences in OSD outcomes, which have not been extensively explored in previous research.

The study also provides new insights into the relationship between pain severity and KOOS subscale scores. The significant negative correlations between pain ratings and KOOS subscales suggest that higher levels of pain are associated with worse knee-related outcomes. This finding underscores the importance of effective pain management strategies in improving the functional capacity and quality of life of individuals with OSD. Previous studies have emphasized the role of physical therapy, activity modification, and NSAIDs in managing OSD symptoms [2]. However, the findings of this study suggest that targeted interventions addressing pain severity may be particularly beneficial in improving outcomes for affected individuals.

The significant gender differences in OSD outcomes suggest that females may require more intensive interventions to manage their symptoms effectively. Future studies could explore the underlying factors contributing to these differences, such as anatomical, hormonal, or psychosocial factors. For example, hormonal fluctuations during puberty may influence pain perception and symptom severity in females [15], warranting further investigation. Moreover, this study highlights the need for personalized treatment plans based on pain severity and functional limitations. Future research could evaluate the effectiveness of tailored interventions, such as targeted physical therapy programs or pharmacological treatments, in improving outcomes for individuals with OSD. Additionally, the role of psychological interventions, such as cognitive-behavioral therapy, in managing pain and improving quality of life could be explored. Educational programs aimed at promoting early diagnosis and appropriate management of OSD could help reduce the long-term impact of the condition. Future studies could evaluate the effectiveness of such programs in improving outcomes for affected individuals.

This study has several limitations. One key limitation of this study is that the inclusion/exclusion criteria did not account for participation in high-impact sports, which is a well-established risk factor for OSD. Since OSD is particularly prevalent among adolescents engaged in activities involving repetitive knee stress (e.g., soccer, basketball, and running), the absence of sports participation data may limit the generalizability of findings. Thus, future studies should stratify participants based on athletic involvement to better assess the association between sports activity and OSD severity. Additionally, controlling for exercise intensity and frequency could help identify high-risk groups and inform targeted preventive strategies. The cross-sectional design does not allow for causal relationships between OSD and observed outcomes, and recall bias may introduce inaccuracies. The sampling method used may not be representative of the entire population, and the findings may not be generalizable to other populations or regions. Self-reported data can be subjective and influenced by individual perceptions, cultural factors, and social desirability bias. The study did not assess the psychological impact of OSD, such as anxiety, depression, or fear of movement (kinesiophobia), which can influence pain perception and functional limitations. Moreover, psychological, behavioral, and cultural confounders (e.g., gendered pain perception or access to healthcare) are not deeply addressed. Future studies should consider longitudinal designs, larger and more representative samples, and objective measures of pain and function. Addressing the aforementioned limitations would enhance the understanding of OSD and inform more effective interventions for affected individuals.

## Conclusions

In conclusion, this study provides valuable insights into the prevalence, awareness, and impact of OSD among adolescents and young adults in Saudi Arabia. The findings highlight the significant burden of OSD on affected individuals, particularly in terms of pain severity and functional limitations. The study also identifies important gender differences in OSD outcomes, suggesting that females may require more intensive interventions to manage their symptoms effectively. These findings have important implications for the management and understanding of OSD, emphasizing the need for personalized treatment plans and targeted interventions to improve outcomes for affected individuals. Future research should build on these findings to explore the underlying factors contributing to gender differences in OSD outcomes, and evaluate the effectiveness of tailored interventions in improving the quality of life for individuals with OSD.
